# 14-3-3ζ Interacts with Stat3 and Regulates Its Constitutive Activation in Multiple Myeloma Cells

**DOI:** 10.1371/journal.pone.0029554

**Published:** 2012-01-18

**Authors:** Jia Zhang, Fangjin Chen, Wenliang Li, Qian Xiong, Mingkun Yang, Peng Zheng, Chongyang Li, Jianfeng Pei, Feng Ge

**Affiliations:** 1 Institute of Hydrobiology, Chinese Academy of Sciences, Wuhan, China; 2 Center for Theoretical Biology, Academy for Advanced Interdisciplinary Studies, Peking University, Beijing, China; 3 School of Science and Technology, Tokai University, Tokyo, Japan; Erlangen University, Germany

## Abstract

The 14-3-3 proteins are a family of regulatory signaling molecules that interact with other proteins in a phosphorylation-dependent manner and function as adapter or scaffold proteins in signal transduction pathways. One family member, 14-3-3ζ, is believed to function in cell signaling, cycle control, and apoptotic death. A systematic proteomic analysis done in our laboratory has identified signal transducers and activators of transcription 3 (Stat3) as a novel 14-3-3ζ interacting protein. Following our initial finding, in this study, we provide evidence that 14-3-3ζ interacts physically with Stat3. We further demonstrate that phosphorylation of Stat3 at Ser727 is vital for 14-3-3ζ interaction and mutation of Ser727 to Alanine abolished 14-3-3ζ/Stat3 association. Inhibition of 14-3-3ζ protein expression in U266 cells inhibited Stat3 Ser727 phosphorylation and nuclear translocation, and decreased both Stat3 DNA binding and transcriptional activity. Moreover, 14-3-3ζ is involved in the regulation of protein kinase C (PKC) activity and 14-3-3ζ binding to Stat3 protects Ser727 dephosphorylation from protein phosphatase 2A (PP2A). Taken together, our findings support the model that multiple signaling events impinge on Stat3 and that 14-3-3ζ serves as an essential coordinator for different pathways to regulate Stat3 activation and function in MM cells.

## Introduction

The 14-3-3 proteins are a family of highly conserved, ubiquitously expressed regulatory molecules and seven isoforms, designated δ, η, γ, ε, θ, β and ζ, have been described previously [1], [2]. The 14-3-3 proteins are known for their ability to bind a plethora of client proteins, mostly through a phosphorylated serine or threonine motif [3], [4]. Because 14-3-3 interactions are primarily phosphorylation- dependent, the 14-3-3 proteins have been tightly integrated into the central phosphor-relay regulatory pathways that form the core of vital signal transduction pathways. Through regulated interactions with crucial signaling mediators, 14-3-3 controls diverse cellular responses ranging from signal transduction, cell cycle progression, metabolism, oncogenesis and apoptosis [5]. The 14-3-3 proteins have raised to a position of integrators of diverse signaling cues that impact cell fate and cancer development [5]. In general, 14-3-3 proteins play a role in promoting survival and repressing apoptosis [6]. However, individual 14-3-3 proteins may have unique functions in certain physiological contexts and might selectively affect distinct aspects of the carcinogenic process [7]. Especially, involvement of 14-3-3ζ in multiple signaling pathways has been reported and activities of various signaling mediators are differentially regulated by 14-3-3ζ *via* direct physical association [8]. However, whether 14-3-3ζ also regulates the signal transducers and activators of transcription (Stat) family was unknown. In the course of our search for proteins that interact with 14-3-3ζ, for the first time, we found that Stat3 is one of the novel 14-3-3ζ interacting proteins [9].

The Stat proteins are a conserved family of transcription factors implicated in regulating processes such as inflammation, survival, proliferation, metastasis, angiogenesis, and chemoresistance of tumor cells [10]. One of these members, namely Stat3, is ubiquitously expressed and is functionally involved in regulating cell proliferation, differentiation and cell survival [11]. In many cancer cells, Stat3 signaling has been recognized as a pivotal pathway supporting survival and growth [12], [13], [14]. Stat3 is often constitutively active in many human cancer cells including multiple myeloma (MM), leukemia, lymphoma, and solid tumors [12], [15]. The Stat3 signaling is modulated, both positively and negatively, by its interaction with numerous other proteins, and crosstalk occurs with various other signaling cascades, including the NF-κB, AP-1 or PI-3K pathways [16]. We made the hypothesis that the physiological interaction between 14-3-3ζ with Stat3 might contribute to the cooperation and/or coordination of their functions in the control of numerous intracellular signaling and regulatory pathways in MM cells.

The purpose of the present study was to further elucidate the molecular mechanisms underlying the regulation of Stat3 pathway and to contribute to a better understanding of the cross-talk between 14-3-3ζ and Stat3 signaling in MM cells. In this study, evidence is provided that 14-3-3ζ interacts with Stat3 in a phosphorylation-dependent manner and the phosphorylated Ser727 is necessary for 14-3-3ζ binding. Of note, 14-3-3ζ is required for nuclear translocation, optimal DNA-binding and transcriptional activity of Stat3. Furthermore, our results indicated that 14-3-3ζ is involved in the regulation of PKC activity and protection of Ser727 dephosphorylation from protein phosphatase 2A (PP2A) in U266 cells. Thus, our findings have important implications in the understanding of the mechanisms that regulate Stat3 activity and function in MM cells.

## Results

### 14-3-3ζ Interacts with Stat3 Protein

**Figure 1:**
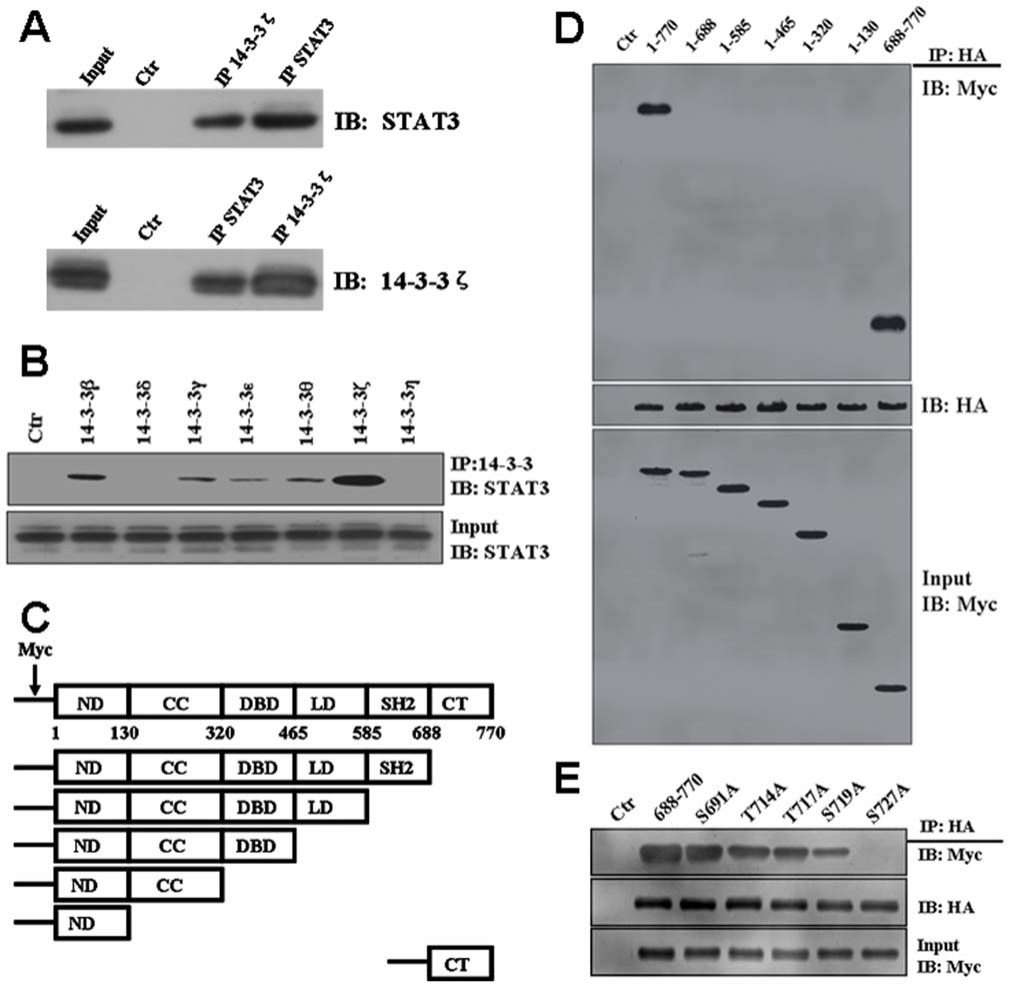
14-3-3ζ is a Stat3-interacting protein. (A) Immunoprecipitation assays of 14-3-3ζ and Stat3 proteins were carried out in U266 cells as described in Materials and Method section. No band of Stat3 was observed in the IgG negative control (Ctr). (B) Interaction of Stat3 with 14-3-3 isoforms. (C) Schematic illustration of Stat3 domains and their truncations. ND: N-terminal domain, CC: coiled-coil domain, DBD: DNA binding domain, LD: linker domain, SH2: SH2 domain and CT: C-terminal. (D) Identification of the Stat3 domains interacting with 14-3-3ζ. Myc-Stat3 domain truncation mutants were transfected with HA-14-3-3ζ in U266 cells. An empty vector was used as a control. Anti-HA immunoprecipitates were analyzed by Western blot with antibodies to Myc (upper panel) or HA (middle panel). The expression of Myc-Stat3 is shown in the bottom panel. (E) Stat3 C-terminal mutants binding to 14-3-3ζ. Experiments were performed in triplicate and representative data are shown.

We had previously reported an interaction between 14-3-3ζ and Stat3 in U266 cells [9]. We further confirm this association between endogenously expressed proteins by using reciprocal immunoprecipitation. As shown in Figure 1A, Stat3 was detected in the 14-3-3ζ immune complex and the U266 cell lysate (Input) but not in the normal rabbit IgG control (Ctr). Furthermore, reverse immunoprecipitation assay using specific antibodies for Stat3 followed by Western blotting with 14-3-3ζ confirmed their binding to 14-3-3ζ. Thus, compelling evidence shows that 14-3-3ζ interacts with Stat3 in U266 cells. Seven 14-3-3 isoforms are expressed in U266 cells [9]. To determine whether Stat3 also interacts with other 14-3-3 isoforms, U266 cell lysates were immunoprecipitated using 14-3-3 isoform specific antibodies [9] and the co-precipitated Stat3 was detected by Western blot. The results showed absence of binding to rabbit IgG control (Ctr) and 14-3-3δ, η; weak binding to γ, ε, θ; moderate binding to β and strong binding to ζ (Fig. 1B). We have shown that several 14-3-3 isoforms (14-3-3β, ε, γ, η, θ) were co-purified with 14-3-3ζ [9], which is in accordance with previous reports showing hetero-dimerization between different 14-3-3 isoforms [17], [18]. Therefore, our results demonstrated that Stat3 can interact with 14-3-3ζ directly and also suggested that Stat3 can bind with other mammalian 14-3-3 isoforms in a redundant manner or through binding to 14-3-3ζ.

### The C-terminal domain of Stat3 Is Responsible for Its Association with 14-3-3ζ

To identify which region of Stat3 is involved in this interaction, we generated Stat3 truncated mutants, successively deleting Stat3 domains from the C terminus (Fig. 1C), and analyzed their ability to interact with 14-3-3ζ. These truncated Stat3 constructs were transfected with 14-3-3ζ into U266 cells. Co-IP experiments revealed that Stat3 C-terminal region (amino acids 688 to 770) retained 14-3-3ζ interaction whereas all other truncated mutants failed to co-IP with 14-3-3ζ (Fig. 1D). These results indicate that the C-terminal tail of Stat3 is responsible for interaction with 14-3-3ζ.

### Serine 727 of Stat3 Is Essential for 14-3-3ζ Binding

A major function of 14-3-3 proteins is to bind to proteins with phosphorylated serine and threonine residues [19], [20], [21]. Since we had demonstrated an association between C-terminal region (amino acids 688 to 770) of Stat3 and 14-3-3ζ, we wanted to determine whether the association between Stat3 and 14-3-3 proteins required phosphorylation. Mass spectrometric analysis identified several phosphorylation sites on the C-terminal tail of Stat3, including S691 [22], T714 [23], [24], T717 [24], S719 [25] and S727 [26], [27], [28], [29]. To find the phosphorylation site that is involved in binding to 14-3-3ζ, myc-Stat3 (688–770) or mutants (S691A, T714A, T717A, S719A and S727A) plasmids were generated and transfected into U266 cells, along with the HA-14-3-3ζ plasmids. Total cell lysates were analyzed by anti-HA IP followed by myc Western blot. The results showed that Stat3 binding to 14-3-3ζ was abrogated by the S727A mutation (Fig. 1E). These results suggested that phsophorylated Ser727 (pSer727) is the major binding site for 14-3-3ζ.

### Modeling of the 14-3-3ζ/Stat3 Complex Allows Rationalization of the Results

**Figure 2:**
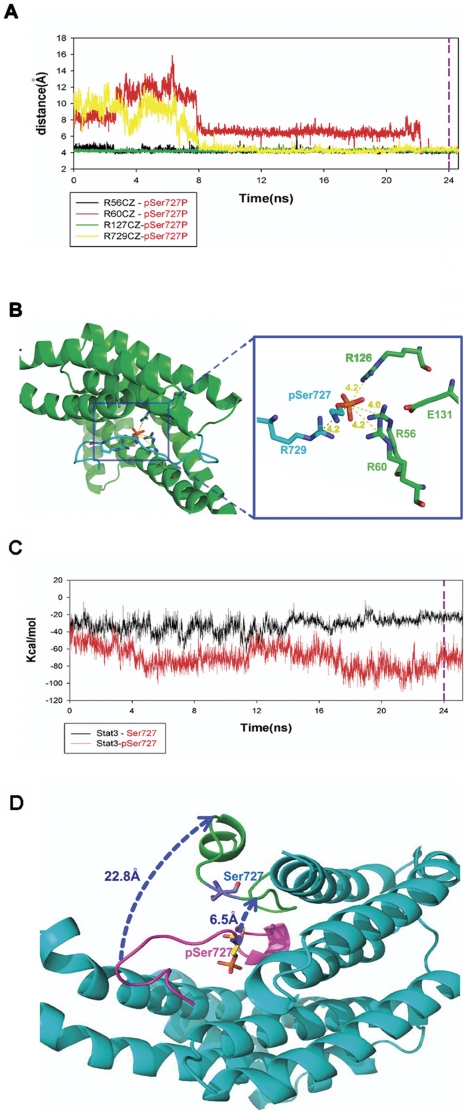
Structural models of 14-3-3ζ in complex with Stat3. (A–B) The crucial amino acids of 14-3-3ζ interacting with phosphorylated Stat3 peptide. Distance of the zeta carbon atoms of R56, R60, and R127 of 14-3-3ζ between the phosphorus atom of the phosphorylated Stat3 peptide after 24 ns MD simulations of balance were shown. (C) Binding free energy change and the structural movements of the Stat3 peptide after dephosphorylation. Differences of the binding free energies of 14-3-3ζ with phosphorylated and dephosphorylated Stat3 peptides. (D) Starting geometry of dephosphorylated Stat3 peptide (purple), and the dissociated dephosphorylated Stat3 peptide after 24 ns MD simulation (green). The 14-3-3ζ is shown in cyan.

To rationalize our results we analyzed the structures of the 14-3-3ζ/Stat3 complex. A number of 14-3-3ζ-partner (most are short peptides) complex structures were solved and available in the PDB database (Table S1). In the complex structures, partners interacting with 14-3-3ζ contain phsophorylated serine or threonine, which bind to positive charged residues R56 and R127, and R60 of 14-3-3ζ. So R56, R127 and R60 are supposed to be the possible binding sites of the pSer727 of Stat3. In all the 14-3-3ζ partner peptides listed in Table S1, a positive charged residue (R or K) which is 1-3 residues away in the N-terminal direction from the phsophorylated site is found. This positive residue is proposed to interact with the phsophorylated site too, helping to stabilize the interaction of 14-3-3ζ R56, R127 and R60 with the phsophorylated site. Stat3 is different to all the above peptides as its positive residue (R) is in the C-termical direction from the phsophorylated site (pSer727). For simplicity of MD simulation, we used a 17-peptide (CSNTIDLPMpSPRTLD SL) of Stat3 (referred to as Stat3 peptide in the following text) that is critical for the binding to represent Stat3. We constructed the starting structure of the Stat3 peptide by residue mutation using 1IB1 as template and then MD simulations with AMBER10 program were carried out to simulate the interaction between 14-3-3ζ and Stat3 peptide with phsophorylated Ser727 (referred to as pSer727 Stat3 peptide in the following text). After more than 24 ns simulation, the system balanced and the key residue pSer727, form four stable salt bridges with R56, R60, R127 of 14-3-3ζ and with R729 of Stat3 peptide itself. All distances between the arginine CZ atoms and the pSer727 phosphorous atom are nearly 4 Å (Fig. 2A and 2B). Then the 24 ns structure was stripped from the MDs trajectory as the starting geometry for further simulations. A 14-3-3ζ/Stat3 peptide complex was constructed by dephosphorylation of the pSer727 of the 14-3-3ζ/pSer727 Stat3 peptide complex. Both 14-3-3ζ/Stat3 peptide complex and 14-3-3ζ/pSer727 Stat3 peptide complex were subject to further MD simulations. The further MD simulations show that without phsophorylated Ser727 Stat3 peptides moves away from the binding region (Video S1). The interaction energies of 14-3-3ζ with Stat3 peptide and pSer727 Stat3 peptide are shown in Figure 2C. After 24 ns simulations the interaction energy of 14-3-3ζ with pSer727 Stat3 peptide (about −80 KCal/mol) is more than three times lower than that with Stat3 peptide (about −20 KCal/mol), which means a significant binding affinity decrease after Ser727 dephsophorylation. The Stat3 peptide also moves away from the interaction region for about 6.5 Å to 22.8 Å (Fig. 2D). Notably, for most of the simulation time, pSer727 and R729 of Stat3 peptide are combined together, which proved our previous suppose that R729 helps to stabilize the interaction of 14-3-3ζ's R residues with pSer727.

### 14-3-3ζ regulates Stat3 transcriptional activity via Ser727

**Figure 3:**
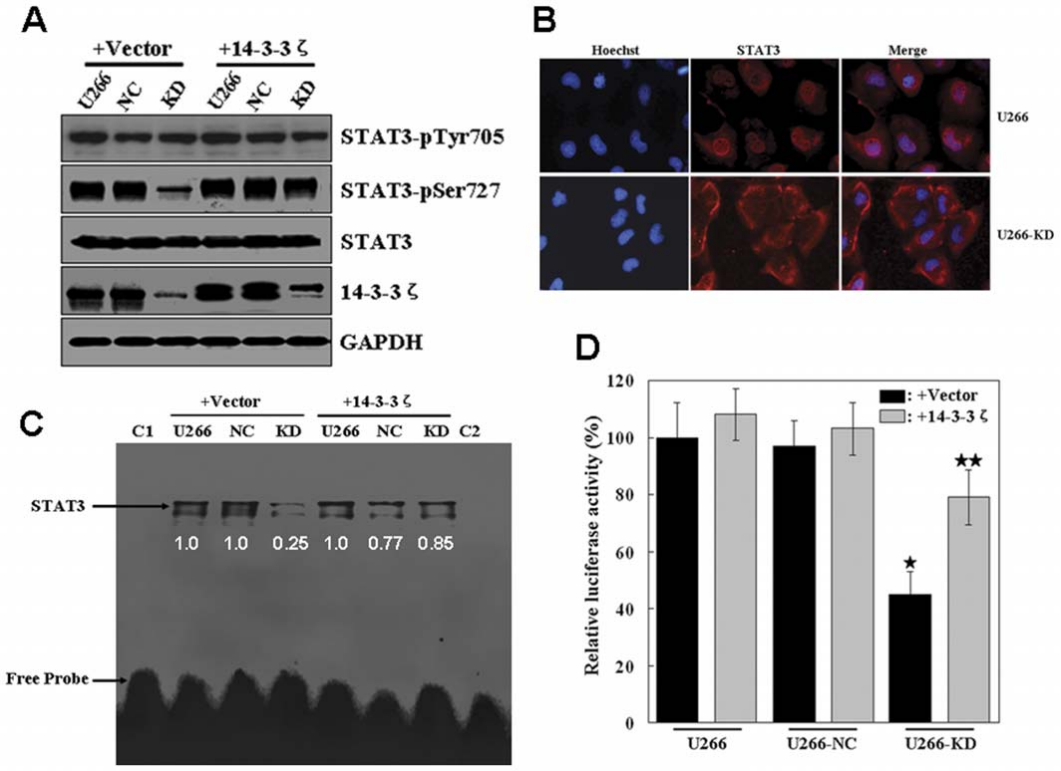
Effect of 14-3-3ζ knock down on Stat3 activity. (A) Effect of 14-3-3ζ knock down on Stat3 activity in U266 cells. U266, 14-3-3ζ knockdown U266 cell line (U266-KD) and its negative control cell line (U266-NC) cells were transfected with 14-3-3ζ plasmid or blank vector. Phosphorylation of Stat3 Tyr705 and Ser727 was detected by the specific anti-phospho-Tyr705 and anti-phospho-Ser727 antibodies, and the blot was stripped and reprobed with anti-Stat3 antibody. The blot was also probed by GAPDH antibody to serve as loading controls. (B) Influence of 14-3-3ζ knock-down on Stat3 nuclear translocation. The subcellular localization of Stat3 in U266-NC and U266-KD cells was monitored by using fluorescence microscopy. (C) 14-3-3ζ knock down inhibits constitutively active Stat3 in U266 cells. U266, U266-NC and U266-KD cells were transfected with 14-3-3ζ plasmid or blank vector as indicated. Nuclear extracts prepared from these cells were incubated with hSIE probe and analyzed by EMSA. Lane 1 (C1), hSIE oligonucleotide only; lane 8 (C2), U266 nuclear extracts+100×excess unlabeled hSIE oligonucleotide. Relative expression of Stat3 (normalized to U266 samples) was determined using image densitometry. (D) Reporter constructs were cotransfected into U266, U266-NC and U266-KD cells together with expression vectors encoding 14-3-3ζ or blank vector as indicated. Luciferase activity in the cells was analyzed by dual-luciferase assay. *Significantly different compared to the U266 and U266-NC cells (*p*<0.01). **Significantly different compared to U266-KD cells transfected with blank vector (*p*<0.01). Experiments were performed in triplicate and representative data are shown.

Stat3 is constitutively activated in U266 cells [12] and various primary tumors and tumor cell lines [30]. Transcriptional activity of Stat3 is controlled by phosphorylation on Ser727 and Tyr 705, followed by dimerization and nuclear translocation. Evidence indicates that cooperation of both tyrosine and serine phosphorylations is necessary for full activation of Stat3 [30], [31]. To investigate the level of Stat3 signaling the 14-3-3ζ acts on, we studied whether 14-3-3ζ knockdown interferes with activation of Stat3, that is, phosphorylation of tyrosine 705 and serine 727. In our previous work, we developed the 14-3-3ζ knockdown U266 cell line (U266-KD) and its negative control cell line (U266-NC) [9]. As shown in Figure 3A, compared with parental U266 and U266-NC cells, Stat3 phosphorylation at S727 was clearly inhibited by 14-3-3ζ depletion, meanwhile phosphorylation at Y705 and total Stat3 protein levels were not affected. Our results prompted us to further investigate the effects of overexpression of 14-3-3ζ in U266 cells. We transiently transfected U266-KD, U266-NC and parental U266 cells with an expression vector containing a cDNA encoding human 14-3-3ζ or a blank vector as a control. The overexpression of 14-3-3ζ was confirmed by Western blotting (Fig. 3A). As shown in Figure 3A, compared with the cells transfected with the control vector, transient expression of 14-3-3ζ increased the Stat3 phosphorylation at S727 in U266-KD cells. The results showed that 14-3-3ζ knockdown inhibited S727 phosporylation of Stat3. Because phosphorylation causes dimerization of Stat3 and then nuclear translocation [12], we therefore next studied whether 14-3-3ζ knock down might influence this translocation process by immunocytochemistry. In U266 cells, Stat3 preferentially localized to the nucleus, as apparent by Hoechst staining (Figure 3B). Depletion of 14-3-3ζ prevented nuclear translocation of Stat3, as was expected from its inhibitory effect on phosphorylation of Stat3 (Fig. 3B). When Stat3 is translocated to the nucleus, it binds to the DNA, an event that in turn regulates gene transcription [12]. Therefore, we monitored the activity of Stat3 dimers inside the nucleus by assessing the Stat3 DNA binding ability in the presence or absence of 14-3-3ζ. Relative expression of Stat3 was determined using image densitometry. As shown in Figure 3C, the DNA-binding ability of Stat3 in the U266-KD cells was significantly reduced (Fig. 3C, lane 4). With the overexpression of 14-3-3ζ, the DNA-binding ability of nuclear Stat3 was increased significantly in the U266-KD cells (Fig. 3C, lane 7). These data indicate that 14-3-3ζ is required for optimal DNA binding of Stat3 to its DNA response element. Next, we further measured the effects of 14-3-3ζ on Stat3 activity in luciferase reporter gene assays. As shown in Figure 3D, knock down of 14-3-3ζ in U266 cells yielded a significant decrease in the Stat3-dependent relative luciferase activity, while overexpression of 14-3-3ζ markedly enhanced Stat3-mediated transcriptional activity in U266-KD cells. Taken together, these data suggest that 14-3-3ζ positively regulates the transcriptional activity of Stat3 and is necessary for Stat3-mediated transcriptional activation in U266 cells.

### PKC activity is compromised in the absence of 14-3-3ζ

**Figure 4:**
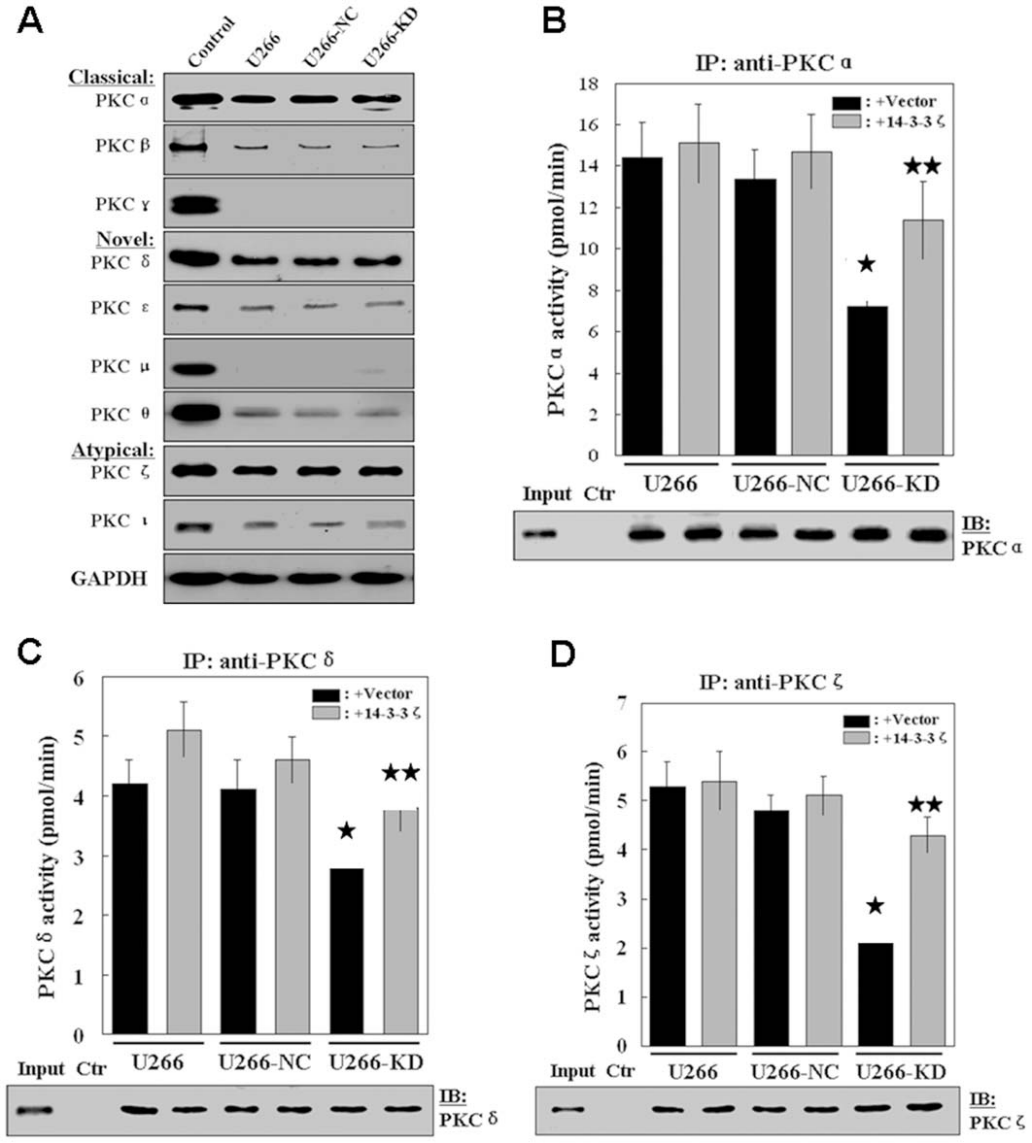
Effects of 14-3-3ζ on PKC activity. (A) PKC isoform expression in U266, U266-NC and U266-KD cells. Cell lysates from rat brain were used as a positive control for PKC expression. (B–D) 14-3-3ζ knock down inhibits PKC isoform kinase activity. PKC isoform activity was determined using PKC immunoprecipitation kinase assays. After transfected with 14-3-3ζ plasmid or blank vector as indicated, equal amounts of whole-cell lysates were immunoprecipitated with PKC α (B), PKC δ (C) and PKC ζ (D) antibodies and immunoblotted with indicated antibodies. IP indicates immunoprecipitation; Ctr, immunoprecipitation with protein A/G Plus beads, whole-cell lysates, and preimmune rabbit serum. *Significantly different compared to the U266 and U266-NC cells (*p*<0.05). **Significantly different compared to U266-KD cells transfected with blank vector (*p*<0.05). Experiments were performed in triplicate and representative data are shown.

It is well established that 14-3-3 proteins function as a protein kinase C (PKC) regulator [32], [33], [34], [35] and 14-3-3ζ activates PKC in vitro [36], [37]. Recently, evidence indicates that PKC interacts with Stat3, phosphorylates Stat3 Ser727, and increases both DNA-binding and transcriptional activity of Stat3 [38]. Therefore, we speculated that 14-3-3ζ might affect Stat3 activation *via* regulating the PKC activity. In MM, PKC isoform expression has been reported in several MM cell lines [39], [40], [41]. Specifically, our results show high expression of PKC α, PKC δ and PKC ζ; low expression of PKCι, PKC β, PKC μ and PKC ε; and absence of PKC θ and PKC γ in U266-NC, U266-KD cells and its parent U266 cell line (Fig. 4A). We therefore focus on PKC α, PKC δ and PKC ζ. We investigated whether 14-3-3ζ knock down affects PKC activity in U266 cells using immunoprecipitation kinase assays. As shown in Figure 4B–D, knockdown of 14-3-3ζ resulted in a significant decrease in the kinase activities of PKC α, PKC δ and PKC ζ (*p*<0.05), whereas the amount of PKCs that was used in each experimental condition was similar, as determined by immunoblotting with specific PKC isoform antibodies. With the overexpression of 14-3-3ζ, the kinase activities of PKC α, PKC δ and PKC ζ were increased significantly in the U266-KD cells (*p*<0.05), compared with the cells transfected with the control vector. Taken together, our results indicate that 14-3-3ζ was involved in the regulation of PKC activity in U266 cells.

### 14-3-3ζ Proteins Protect Stat3 phosphorylation at S727 from PP2A Activity

**Figure 5:**
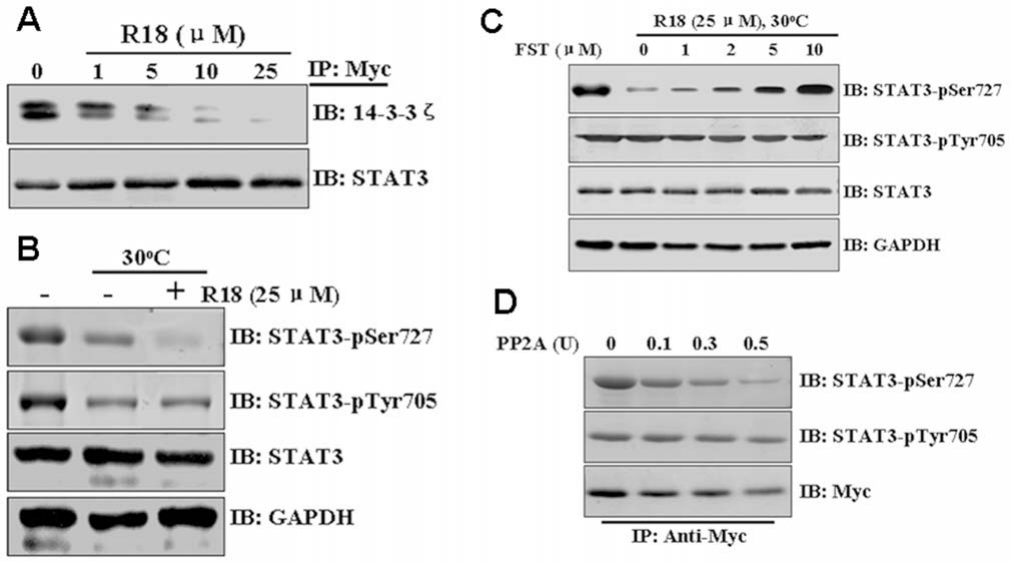
14-3-3ζ protects Stat3 phosphorylation. (A) Competition of R18 peptide for 14-3-3ζ/Stat3 interaction. Immunoprecipitated 14-3-3ζ from U266 cells transfected with Myc-Stat3 was incubated with varying amounts of R18 and analyzed by Western blotting with the indicated antibodies. (B) Total cell lysates were prepared in phosphatase lysis buffer A, and aliquots were either left on ice or dephosphorylated in the presence (+) or absence (−) of 25 µM R18 peptide. Reactions were analyzed by immunoblotting (IB) with the indicated antibodies. (C) Lysates from U266 cells were prepared as in A. Aliquots were either left on ice or dephosphorylated in the presence of 25 µM R18 peptide. Where indicated, FST (1–10 µM) was added on ice for 10 min before initiating dephosphorylation. Reactions were analyzed by Western blotting with the indicated antibodies. (D) Immunoprecipitated Stat3 from U266 cells was treated with the indicated units of purified PP2A enzyme at 30°C for 30 min, and analyzed by Western blotting with the indicated antibodies. All experiments were performed in triplicate and representative data are shown.

It has been reported that 14-3-3 can protect phosphorylated proteins from access by the catalytic unit of the protein phosphatase 2A (PP2A) thus preventing its dephosphorylation [42]. Therefore, we hypothesized that binding of 14-3-3ζ to Ser727 of Stat3 protects it from phosphatase activity. To test this hypothesis, we used a nonphosphorylated peptide, R18, which has been shown to displace 14-3-3 from its phosphorylated binding partners [43], [44]. As predicted, the R18 peptide efficiently displaced 14-3-3ζ from Stat3 (Fig. 5A). Lysates containing phosphorylated Myc-Stat3 were then subjected to an *in vitro* dephosphorylation assay in a buffer compatible with phosphatase but not kinase activity. As shown in Figure 5B, S727 was dephosphorylated when 14-3-3ζ was displaced by R18, suggesting the presence of an active phosphatase (s). Dephosphorylation of Y705, site not implicated in 14-3-3ζ binding, was unaffected by R18 (Fig. 5B). Because previous data suggest that PP2A is the predominant Ser/Thr phosphatase that interacts with Stat3 [45], we addressed its contribution to Ser727 dephosphorylation in cell-free extracts containing FST, a selective inhibitor of PP2A [46]. In the presence of R18, FST inhibited dephosphorylation of Stat3 at S727 in a dose-dependent manner (Fig. 5C). To demonstrate that PP2A has the capacity to directly dephosphorylate Stat3, we performed an *in vitro* phosphatase assay. As shown in Figure 5D, increasing amounts of purified PP2A effectively dephosphorylated Stat3 at S727. Collectively, our findings strongly suggest that the association between Stat3 and 14-3-3ζ protects S727 dephosphorylation from PP2A.

## Discussion

Stat3 activity is tightly regulated by its interacting proteins and multiple signaling cascades and its prolonged activation is associated with various malignancies, including MM [12], [47]. In this study, we reported a novel interaction between 14-3-3ζ and Stat3 in myeloma cells. Through the use of multiple biochemical approaches, we demonstrated that 14-3-3ζ is a *bona fide* Stat3 interacting partner in U266 cells. Phosphorylation of the Ser727 residue in the C terminus of Stat3 mediates the Stat3/14-3-3ζ interaction, and replacement of this crucial residue with phosphorylation-resistant Alanine totally abolishes the protein associations (Fig. 1E). Based on these results, it is possible that 14-3-3ζ interacts only with Ser727-phosphorylated Stat3 in U266 cells. The Stat3/14-3-3ζ interaction leads to an increase of the endogenous Stat3 transcriptional activity, whereas the absence of 14-3-3ζ leads to significant impairment in Stat3 activity (Fig. 3). These results suggest that 14-3-3ζ is a positive regulator of Stat3 activity. Generally, in addition to the phosphorylation of Tyr705, phosphorylation of the Ser727 residue also contributes to the activation of Stat3. While tyrosine phosphorylation plays a key role in all the basic events required for Stat3 activation, such as dimer formation, nuclear translocation and DNA binding, serine phosphorylation is required for its maximal transcriptional activity. Serine phosphorylation, therefore, probably represents a second level in the regulation mechanism through which, with the assistance of 14-3-3ζ, maximal Stat3 transcriptional activity is achieved. 14-3-3ζ may, in fact, serve as a molecular stabilizer, specifically devoted to serine phosphorylation, by converting Stat3 from its ‘primarily activated’ form to ‘optimally activated’ form. This second stage of regulation may involve various events such as enhanced Stat3 DNA binding and coactivator recruitment. Identification of Stat3/14-3-3ζ interaction *via* the Ser727 residue also provides a potential explanation for the long-undetermined mechanism of the serine phosphorylation-mediated enhancement to Stat3 transcription.

**Figure 6:**
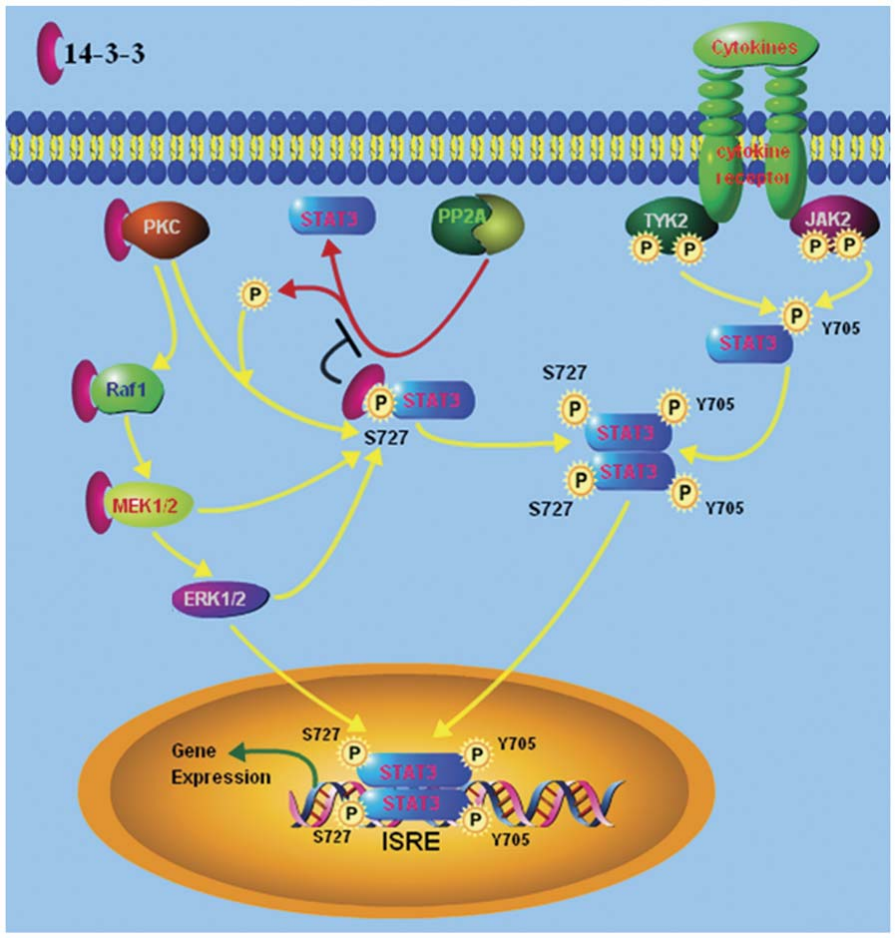
Proposed model of 14-3-3 and Stat3 interaction. The model illustrated that multiple signaling events, including PKC, Raf1, MEK1/2, ERK1/2 and PP2A, impinge on Stat3 and that 14-3-3ζ serves as an essential coordinator for different pathways to regulate Stat3 activity in myeloma cells.

Importantly, it is well established that 14-3-3 proteins are involved in various steps regulating the Stat3 activity, including PKC [32], [33], [34], [35], Raf1 [48], [49], MEK1/2 [50], and PP2A [42]. Consistent with these reports, our results demonstrated that 14-3-3ζ is involved in the regulation of PKC activity (Fig. 4) and the association between Stat3 and 14-3-3ζ protects Ser727 dephosphorylation from PP2A (Fig. 5). These data, along with the results on Stat3/14-3-3ζ interaction, support the model that multiple signaling events, including PKC, Raf1, MEK1/2, ERK1/2 and PP2A, impinge on Stat3 and that 14-3-3 proteins serve as an essential coordinator for different pathways to regulate Stat3 activity in myeloma cells (Fig. 6). As shown in Figure 6, 14-3-3ζ is linked to constitutive activation of Stat3 in myeloma cells. 14-3-3ζ and Stat3, the proteins with oncogenic traits, are important components of development and maintenance of MM.

Recently, 14-3-3ζ has been identified as a prognostic marker and therapeutic target for multiple tumor types [5]. We also reported that inhibition of 14-3-3ζ decreases the activity of some pathways of the MM signaling network and induces apoptosis in MM cells [51]. Therefore, 14-3-3ζ contributes to the maintenance of the MM survival network and pharmacologic inhibition of 14-3-3ζ could be an interesting approach to develop novel therapies for MM. However, targeting 14-3-3ζ may be challenging at the current stage because 14-3-3ζ regulates many important proteins that are essential for homeostasis. Based on our findings that 14-3-3ζ and Stat3 act in concert in the maintenance of the MM signaling, it is worthwhile considering a more specific strategy that target Stat3/14-3-3ζ interaction, as such drugs may spare some of the essential homeostatic functions executed by 14-3-3ζ and Stat3 on their own, while inhibiting their malicious cooperation in cancer cells. This should alleviate the systemic toxicity associated with total 14-3-3ζ or Stat3 inhibition and retain most of their functions but may affect tumor growth. Taken together, the malicious cooperation formed by 14-3-3ζ and Stat3 may serve as the Achilles Heel of MM and many other cancers, providing new opportunities for therapeutic intervention. Experiments testing this hypothesis are ongoing in our laboratory.

## Materials and Methods

### Materials and Antibodies

The R18 peptide, with a sequence of PHCVPRDLSWLDLEANMCLP, was purchased from Biomol International (Plymouth Meeting, PA). Fostriecin (FST), a selective inhibitor of protein phosphatase 2A (PP2A) was purchased from Sigma-Aldrich (Taufkirchen, Germany). The antibodies and sources of the antibodies used in this study were as follows: 14-3-3ζ, 14-3-3ε, 14-3-3σ, 14-3-3θ, 14-3-3β, GAPDH antibodies (Santa Cruz Biotechnology, Santa Cruz, CA), 14-3-3γ, 14-3-3η, Stat3, pY705-Stat3, pS727-Stat3 antibodies (Cell Signaling, Danvers, MA), HA tag, Myc tag antibodies (Genecopoeia, Rockville, MD). Antibodies to PKC isoforms were obtained from BD Biosciences (San Jose, CA).

### Cell Cultures

The human myeloma cell line U266 was purchased from American Type Culture Collections (Rockville, MD). All cells were routinely maintained in RPMI 1640 supplemented with 1% penicillin/streptomycin, 1 mmol/L L-glutamine, and 10% fetal bovine serum at 37°C, 5% CO_2_ in air. Stable 14-3-3ζ knock down (designated as U266-KD) and its negative control (designated as U266-NC) cell lines were generated and maintained as previously described [9].

### Plasmid Constructs and Transient Transfections

Human Stat3 and its truncated isoforms corresponding to amino acids 1–688, 1–585, 1–465, 1–320, 1–130 or 688–770 were cloned into the NH_2_ terminal Myc-tagged pReceiver-M43 expression vector (Genecopoeia, Rockville, MD). Human 14-3-3ζ was cloned into the NH_2_ terminal HA-tagged pReceiver-M06 expression vector.

To create point mutations in the C terminus of Stat3, the expression vector encoding C terminus of Stat3 (688–770) was used as a template. Construction of mutant Stat3 (688–770) cDNAs, where the codons for S691, T714, T717, S719 and S727 are exchanged to Ala, was performed by site-directed mutagenesis using a QuikChange kit (Stratagene, La Jolla, CA) following the manufacturer's instructions. Primers used, with the introduced mutations underlined, were: (Ser691→Ala) 5′-GTCGGCCAGAGGCCCAGGAGCAT-3′; (Thr714→Ala) 5′-CTGTGTGGCACCAAC GACCTGC-3′; (Thr717→Ala) 5′-GTGA CACCAACGGCCTGCAGCAAT-3′; (Ser719→Ala) 5′- CGACCTGCGCCAATA CCATTGAC-3′; (Ser727→Ala) 5′-GACCTGCCGATGGCCCCCCGCAC-3′. All mutant constructs were confirmed by DNA sequence analysis. The plasmids were introduced into U266 cells using the Nucleofector X005 (Amaxa, Cologne, Germany), according to the Optimized Protocol for the U266B1 cell line.

### Immunoprecipitation and Western blot analysis

Protein extracts prepared with lysis buffer (150 mM NaCl, 10 mM HEPES, pH 7.5, 0.2% Nonidet P-40, 5 mM NaF, 5 mM Na_4_P_2_O_7_, 2 mM Na_3_VO_4_, 10 mg/l aprotinin, 10 mg/l leupeptin, and 1 mM PMSF), incubated on ice for 30′, and centrifuged to remove insoluble materials. The BioRad (Hercules, CA) protein assay was used to measure protein concentrations. For immunoprecipitations, cell extract was precleared by Protein A/G Plus beads (Santa Cruz Biotechnology) followed by incubation with primary antibody overnight on a rocker at 4°C. Immune complexes were pulled down by incubating with Protein A/G Plus beads for 4 h at 4°C followed by washing twice with lysis buffer containing 0.1% Triton X-100, and two times with lysis buffer without detergent. Bound proteins were eluted by boiling and analyzed by Western blot. For Western blots, 30 µg of cellular extract was resolved by 10% SDS-PAGE, transferred to nitrocellulose, and probed with appropriate antibodies. These experiments were repeated three times and representative data are shown.

### Preparation of the structures for MD simulations

The crystal structure of the 14-3-3ζ/serotonin N-acetyltransferase complex (PBD ID: 1IB1) was used as the template to construct the 14-3-3ζ/Stat3 peptide complex by mutating the residues on N-acetyltransferase to the corresponding residues of Stat3, with the phosphorylation residues (S for Stat3 and T for N-acetyltransferase) for sequence alignment point. After 24 ns balance with simulations, the pSer727 Stat3 peptide was then used as a template to construct a dephosphorylated Stat3 peptide. All the structures were modeled by using the program LEaP embeded in AMBER10 program with the parm99 AMBER force field [52]. The protonation states of HIS164 were treated as HID (ND1-protonated) form. The systems were neutralized and immersed in octahedral periodic box of TIP3P [53] water molecules with a closeness parameter of 8 Å away from the boundary of any atoms. The AMBER force field parameter for phosphoserine, which were retrieved from the AMBER parameter database (http://www.pharmacy.manchester.ac.uk/bryce/amber/) [54], [55] were used to build the phosphorylated structure. The 14-3-3ζ/pSer727 Stat3 peptide complex system contains 9043 waters and 9 Na^+^ ions with a volume of 361016.092 Å^3^ (oct). The 14-3-3ζ/Stat3 peptide complex system contains 8640 waters and 11 Na^+^ ions with a volume of 349988.538 Å^3^ (oct).

### MD Simulations

The Ewald method [56] was used for the treatment of long range electrostatic interactions and the SHAKE algorithm were used for constraining all bonds involving hydrogen atoms. The non-bond interaction cutoff was set to 8.0 Å. Energy minimization was performed for each solvated complex using the conjugate gradient algorithm, harmonic constraints were applied with a force constant gradually relaxed from 2 kcal/Å. After minimization, all systems were heated up from 0 K to 310 K during 50 ps, then 50 ps density equilibration at 310 K with weak restraints on the complex (2 kcal/Å) was carried out. Production runs were carried out for more than 24 ns at 310 K. An integration time-step of 2 fs was used and structures were saved every 4 ps. The systems were run with constant pressure and temperature (NPT ensemble mode) with periodic boundary conditions. Constant pressure was maintained using the Langevin piston method with a 1 kDa pressure piston, a piston collision frequency of 2 ps-1.

### MM-PBSA Calculations and Analysis

The binding free energy was calculated by the MM/PBSA (Molecular Mechanics/Poisson-Boltzmann Surface Area) [57], [58] method using the MM-PBSA package of the AMBER10 [52], [59]. It needs dynamical sampling of the system in explicit water and also needs to post-process the trajectory of the system from MD production runs. The binding free energy was calculated by using a simple thermodynamic cycle that combines the molecular mechanical and continuum solvent approach PB [60], [61]. We get snapshot every 4 ps from MD trajectory and the binding free energy was calculated according to the equation [62]:
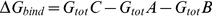
(1)where C, A and B stand for complex, monomer A and monomer B for sake of representation. The free energy of each species was calculated ad follows:
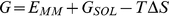
(2)where E_MM_ was the molecular mechanics energy, or enthalpic contribution and were given by internal energy (bonds, angles and dihedrals) (E_int_), electrostatic energy (E_ele_) and van der waals term (E_vdw_):
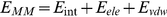
(3)G_SOL_ denoted the salvation free energy which was composed of the polar and nonpolar part. The polar part is the electrostatic contribution to solvation which calculated by solving the linear Poisson Boltzmann equation in a continuum model of the solvent. The nonpolar part accounts for the cost of opening a cavity in the condensed phase, which is related linearly to the solvent accessible surface area [63]. G_SOL_ was calculated according to the equation in AMBER10. TΔS is omitted since for both 14-3-3ζ/pSer727 Stat3 peptide and 14-3-3ζ/Stat3 peptide, the TΔS of them are similar.

### Immunocytochemistry

The U266 and U266-KD cells were grown on sterile glass coverslips using 24-well plates coated with poly-l-lysine (Sigma, USA) and then fixed with 4% paraformaldehyde, permeabilized by 0.1% Triton X- 100. After a brief washing in PBS, slides were blocked with 5% bovine serum albumin for 1 h and then incubated with the anti-Stat3 at a dilution of 1∶500. The cells were then washed three times with PBS and incubated with Texas red-conjugated secondary antibody (Santa Cruz Biotechnology) at a dilution of 1∶500 for 1 h. Finally, the cells were washed with PBS followed by incubation with Hoechst (50 ng/mL) for 15 min and then thoroughly washed again with PBS. The coverslips with stained cells were mounted on glass slides with anti-fade mounting medium and viewed under a fluorescence-microscope (Nikon, Japan). Pictures were captured using a Photometrics Coolsnap CF color camera (Nikon). Experiments were performed in triplicate and representative data are shown.

### Stat3 Luciferase Reporter Assay

U266, U266-NC or U266-KD cells were transfected with a blank or HA-14-3-3ζ plasmid, and a Stat3 firefly luciferase reporter plasmid pStat3-TA-luc (Clontech, Mountain View, CA) and a control Renilla luciferase reporter plasmid pRL-TK (Clontech) in a 3∶1.5∶0.5 ratio using the Nucleofector X005 (Amaxa, Cologne, Germany), according to the protocol described above. 48 hours after transfection, the luciferase activity was determined using a Dual-Luciferase Reporter Assay kit (Promega, Madison, WI) according to the manufacturer's protocol. Experiments were performed in triplicate. Luciferase values were normalized by transfection efficiency as measured by β-galactosidase. All data represent mean values ± s.d. of three independent experiments.

### Electrophoretic Mobility Shift Assay (EMSA) for Stat3-DNA Binding

U266, U266-NC or U266-KD cells were pelleted and washed twice in ice-cold PBS. Nuclear protein extracts were prepared with a nuclear extract kit (Active Motif, Carlsbad, CA) and Stat3-DNA binding activities were assessed by chemiluminescent electrophoretic mobility shift analysis (EMSA) Kit (Pierce, Rockford, IL), according to the manufacturer's protocol. Briefly, nuclear protein extracts (10 µg) were incubated in a final volume of 20 µL of 10× binding buffer, 50% Glycerol, 100 mM MgCl2, 1 µg/µL Poly (dIdC), 1% NP-40 with the biotin end-labeled high-affinity sis-inducible element (hSIE) probe (5′-CTTCATTTCCCGTAAATCCCTAAAGCT- 3′) derived from the c-fos gene promoter, as described [33], [34] for 30 min at RT and terminated by adding 2.0 µL of 10× loading buffer (0.2% (w/v) bromophenol blue and 0.2% xylene cyanol containing 10% (v/v) glycerol). Assays were loaded onto native 5% polyacrylamide gels that were pre-electrophoresed for 60 mins in 0.5× Tris borate/EDTA buffer, resolved at 100 V, and transferred onto nylon membranes (Hybond™-N+, Amersham) in 0.5× Tris borate/EDTA buffer at 100 V for 30 mins. DNA was cross-linked (120 mJ/cm^2^) and detected using HRP-conjugated streptavidin chemiluminescence. For competition assays, nuclear extracts containing equal amounts of total protein were incubated with 100-fold molar excess of unlabeled hSIE probe. The immunoblots were scanned, and densitometric analysis was performed using the public domain NIH Image program ImageJ (developed at the U.S. National Institutes of Health and available on the Internet at http://rsb.info.nih.gov/nih-image/). This experiment was repeated three times and representative data are shown.

### Measurement of PKC Activity

PKC activity was determined in PKC immunoprecipitate using a PKC assay kit (Millipore, Billerica, MA) according to the manufacturer's instructions. The assay kit is based on phosphorylation of a specific substrate peptide (QKRPSQRSKYL) using the transfer of the γ-phosphate of adenosine-5′-[^32^P] triphosphate ([γ-^32^P] ATP) by PKC kinase. The phosphorylated substrate is then separated from the residual [γ-^32^P] ATP using P81 phosphocellulose paper and quantitated by using a Beckman LS 6500 scintillation counter (Brea, CA). Endogenous phosphorylation of proteins in the sample was determined by substituting the assay dilution buffer for the substrate mixture. To assure that equal amounts of PKC were used in the assay, immunoprecipitates were denaturated, eluted, separated by 10% SDS-PAGE, electrophoretically transferred, and immunoblotted with PKC antibody. This experiment was repeated three times and representative data are shown.

### In Vitro Stat3 Dephosphorylation Assay

U266 cells transiently transfected with Myc-Stat3 were washed twice with PBS. Cell extracts were prepared in phosphatase assay buffer A (50 mM Tris-Cl, pH 7.5, 150 mM NaCl, 1 mM EDTA, 0.25% Nonidet P-40, 1 mM phenylmethylsulfonyl fluoride, 10 µg/ml aprotinin, and 10 µg/ml leupeptin, which was also supplemented with one tablet of serine and cysteine protease inhibitor per 10 ml of buffer). The buffer lacked serine/threonine phosphatase inhibitors, and the cell extracts were maintained on ice. Extracts were cleared of cellular debris by centrifugation at 14,000× *g* for 5 min at 4°C. For competition assay, aliquots of clarified lysates containing equal amounts of protein were immunoprecipitated with anti-Myc antibody as described above and incubated with various concentrations of R18 peptide for 60 min on ice. For dephosphorylation assay, aliquots of lysates were incubated either with or without 25 µM R18 peptide. Where indicated, FST was added to the appropriate aliquots on ice for 10 min before initiation of dephosphorylation. Dephosphorylation was performed by incubating lysates for 30 min at 30°C with intermittent mixing before terminating the reaction by boiling in 4×Laemmli reducing sample buffer. For dephosphorylation of Stat3 by PP2A, four aliquots of RIPA buffer (50 mM Tris-HCl, pH 7.4, 150 mM NaCl, 1% [V/V] NP-40, 0.5% [W/V] sodium deoxycholate, 0.1% [W/V] SDS, and 5 mM EDTA) extracts from U266 cells transfected with Myc-Stat3, equalized for protein concentration and volume, were subjected to immunoprecipitation with anti-Myc antibody. Three of the anti-Myc immunocomplexes containing Stat3 were resuspended in phosphatase lysis buffer B [20 mM 3-(*N*-morpholino)propanesulfonic acid, pH 7.5, 150 mM NaCl, and 14.4 mM β-mercaptoethanol] supplemented with Complete (Roche, Indianapolis, IN) but no phosphatase inhibitors and subjected to treatment with the indicated units of purified PP2A purified enzyme (Upstate, Temecula, CA) for 30 min at 30°C with intermittent mixing. The remaining immunocomplex was incubated in phosphatase lysis buffer B alone. The reactions were terminated by boiling in 4×Laemmli reducing sample buffer. The proteins were resolved by SDS-PAGE and then immunoblotted with the appropriate antibodies. This experiment was repeated three times and representative data are shown.

### Statistical Analysis

Data are expressed as the mean ± standard error of the mean from at least three separate experiments performed in triplicate, unless otherwise noted. Statistical analysis was performed using a two-tailed Student's *t*-test. Results were considered significant if *p* values were less than 0.05.

## Supporting Information

Table S1 **Sequences of 14-3-3ζ interaction peptides in Protein Data Bank. Red letter stands for the residue which interacts with phosphorylated residue (blue letter).**
(DOC)

Video S1 **The movements of 14-3-3ζ (cyan), the Ser727 phosphorylated STAT3 peptide (purple) and the dephosphorylated STAT3 peptide (green) during MD simulations were shown in the movie.** As shown in the video, the dephosphorylated STAT3 peptide moves away from the binding region and the residue pSer727 of phosphorylated STAT3 peptide forms four stable salt bridges with R56, R60, R127 of 14-3-3ζ and with R729 of phosphorylated Stat3 peptide itself.
(AVI)
