# Supplementary material for: 14-3-3ζ Interacts with Stat3 and Regulates Its Constitutive Activation in Multiple Myeloma Cells
Source: PLoS One. 2012 Jan 18;7(1):e29554. doi: 10.1371/journal.pone.0029554 (PMC3261159; doi:10.1371/journal.pone.0029554)
Supplement: Table S1 — Sequences of 14-3-3ζ interaction peptides in Protein Data Bank. Red letter stands for the residue which interacts with phosphorylated residue (blue letter). (DOC) [file pone.0029554.s001.doc]

| PDB ID | Sequence of Peptide **^a)^** |
| --- | --- |
| 3NKX | RSTpSTPNVH |
| 1QJA | RLYHpSLPA |
| 2C1J | ARALYpSTGGK |
| 2C1N | ARKpSTGGK |
| 2V7D | KSApTTTVM |
| 3CU8 | RSTpSTPNVH |
| 1IB1 | PGSPGQRRHpTLPANEFR…. |
| Stat3 peptide | CSNTIDLPMpSPRTLDSL |
